# Global Research Hotspots and Trends in Advances of the Ilizarov Technique: A Bibliometric Mapping

**DOI:** 10.1111/os.14325

**Published:** 2025-01-29

**Authors:** Xinxin Wang, Fei Lu, Wenxia Wang, Xiaoyan Zhi

**Affiliations:** ^1^ Department of Nursing The Second Affiliated Hospital of Zhejiang University School of Medicine Zhejiang China

**Keywords:** bibliometrics, CiteSpace, Ilizarov, visual analysis, web of science

## Abstract

**Objective:**

An updated bibliometric analysis is needed to address the lack of comprehensive understanding of Ilizarov technique's research trends and hotspots, fostering collaboration and technology adoption. CiteSpace was utilized to perform co‐citation analyzes on authors, countries, institutions, journals and cited journals, authors and cited literature, along with keywords. This approach aimed to identify leaders, collaborating institutions, and research hotspots associated with the Ilizarov technique, while also predicting future development trends.

**Methods:**

Data relevant to Ilizarov technologies from 1994 to 2023 were extracted from Science Net's core collection. Excel was utilized to develop an exponential function for forecasting annual publication numbers. CiteSpace V5.5 was used to conduct co‐citation analyzes, which included authors, countries (regions), institutions, journals, citation journals, authors, citations, and keywords. Burst detection algorithms were applied to analyze countries (regions), institutions, and keywords, with keyword clustering achieved using the logarithmic likelihood ratio.

**Results:**

A total of 2030 studies were collected, with annual publications on the Ilizarov technique fitting an exponential model *Y* = 3E‐37e^0.0439*x*^ (*R* ^2^ = 0.7979). Morasiewicz Piotr from the University of Opole in Poland emerged as the most prolific author. The leading countries included the USA and China, and notable institutions included the Egyptian Knowledge Bank and the Ilizarov National Scientific Center for Restorative Traumatology and Orthopaedic. Research outputs appeared primarily in orthopedics and surgery, with a focus on keywords such as management, the Ilizarov technique, external fixation, distraction osteogenesis, reconstruction, and the Ilizarov method.

**Conclusions:**

Based on current global trends, the number of publications in the Ilizarov field will continue to increase. Future studies will likely place more emphasis on advancing application concepts and device development.

## Graphical abstract

The figure is a map of the authors' cooperative relationships. High‐impact authors in the Ilizarov technique field were identified using CiteSpace for co‐citation analysis, which produced a distribution map of co‐cited authors comprising 923 nodes and 1134 connections. According to co‐citation analysis theory, a prominent node indicates a key figure influencing the entire co‐cited network. The top five cited authors are Morasiewicz Piotr (26 citations) from the University of Opole in Poland, Aihemaitijiang Yusufu (26 citations) and Maimaiaili Yushan (13 citations) from The Fourth Affiliated Hospital of Xinjiang Medical University in China, and S Robert Rozbruch (21 citations) and Austin T Fragomen (20 citations) from the Hospital for Special Surgery in the USA.

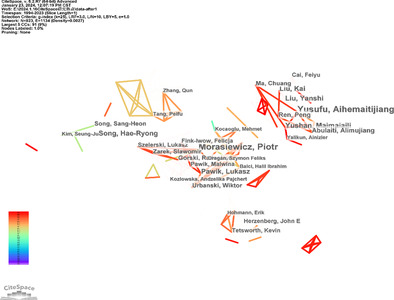

**Abbreviations**
3D: Three‐Dimensional
AI: Artificial Intelligence
BMPs: Bone Morphogenetic Proteins
BMSCs: Bone Marrow Mesenchymal Stem Cells
CAD: Computer‐Aided Design
Clin Orthop Relat R: Clinical Orthopaedics and Related Research
ECM: Extracellular Matrix
IF: Impact Factor
IGF‐1: Insulin‐like Growth Factor 1
Injury: Injury International Journal of the care of the injured
J Bone Joint Surg AM: Journal of Bone and Joint Surgery American Volume
J Bone Joint Surg BM: Journal of Bone and Joint Surgery British Volume
J Orthop Trauma: Journal of Orthopaedic Trauma
J Pediatr Orthoped: Journal of Pediatric Orthopaedics
J trauma: Journal of Orthopaedic Trauma
LLR: Logarithmic Likelihood Ratio
RANK: Receptor Activator of Nuclear Factor‐κB
RANKL: Receptor Activator of Nuclear Factor‐κB Ligand
TGF‐β: Transforming Growth Factor‐β
The USA: The United States of America
WOS: Web of Science

## 1 Introduction

The Ilizarov technique, pioneered by the former Soviet doctor Ilizarov, stands as a widely adopted treatment method globally for patients dealing with bone infection, delayed bone union, and nonunion [1]. The incidence of these conditions in patients with various types of fractures ranges from 0% to 80% [1, 2]. This technique employs distraction osteogenesis principles, where a free bone segment, aided by external fixed scaffolds, is gradually moved toward the bone defect site, stimulating bone tissue regeneration within the elongated space [3]. Ilizarov technology, with its foundation in distraction osteogenesis, represents a significant advancement in orthopedic treatment technology [4]. As research continues, new hotspots and evolving trends in Ilizarov technique development underscore the importance of studying its trajectory and underlying mechanisms.

CiteSpace, a visual knowledge mapping tool based on the Java language, was developed by Chaomei Chen of Drexel University. This tool operates on the principle of “co‐citation analysis theory” and utilizes the “Pathfinder algorithm” to quantitatively analyze literature within specific research domains, thereby identifying key pathways in knowledge evolution in these fields [5]. Widely applied, CiteSpace has been instrumental in studying disease hotspots and trends, contributing to the scientific understanding and management of conditions such as spinal diseases [6] and electrochemiluminescence sensing technology [7, 8]. As a leading international tool for literature visualization and analysis, CiteSpace employs complex network analysis suitable for dynamic and time‐sensitive studies. It generates knowledge graphs that depict the development of scientific fields, providing insights into key literature, emerging research areas, and frontier directions [9].

In 1994, Hetao Xia established the Beijing External Fixation Technology Research Institute and collaborated with experts such as Sihe Qin, devoting themselves to the research and development of circular external fixators tailored to the physical characteristics of the Asian population [10]. This initiative marked a significant step in the localization of Ilizarov technology and constituted a major milestone in technological innovation and development within the field of orthopedics. Despite dedicated efforts to localize the Ilizarov technique, a lack of comprehensive understanding of its research trends and hotspots hinders collaboration and technology adoption. An updated bibliometric analysis is needed to identify research hotspots, foster collaboration, advance technology adoption, and contribute to the continuous development of the Ilizarov technique. Hence, this study gathered research data on the Ilizarov technique beginning in 1994 and employed CiteSpace for co‐citation analyzes involving authors, countries (regions), institutions, journals and citation journals; authors and citations; and keywords.

By integrating co‐citation frequency, centrality measures, and relevant references, this study investigated emerging research hotspots and development trends via the Ilizarov technique, providing an updated bibliometric analysis. The findings offered a valuable reference for future exploration in this field, fostering collaboration among researchers, advancing the adoption of new technologies, and addressing associated challenges. The study aimed to provide a comprehensive overview of the Ilizarov technology research field from two perspectives: (1) general characteristics, such as publication volume, countries of contribution, and core journals; and (2) substance content, including developmental frameworks and research frontiers. The bibliometric analysis aimed to elucidate the research field's current state and emerging trends regarding the Ilizarov technique.

## 2 Materials and Methods

### 2.1 Source

Using the research topics “Ilizarov method,” “Ilizarov technique,” or “Ilizarov treatment,” relevant research data were retrieved from the Web of Science core collection, which spans from 1 January 1994 to 31 December 2023. The literature type was unrestricted. To ensure that no crucial data was overlooked, two researchers from our team independently examined the information regarding the Ilizarov method at the same time and, after discussion, determined the final literature to be included in the study. The results were downloaded in plain text format, including “Full Record and cited References,” capturing annual publication counts in specific journals, research types, and journal citation metrics.

### 2.2 Descriptive Analysis and Fitting Function Construction

The annual publication counts, types of research, and journal publication metrics were imported into Excel 2021. Annual publication trends were analyzed using Excel, applying exponential, linear, logarithmic, and quadratic functions, with selection based on the highest correlation coefficient *R* ^2^. Research types were classified based on CiteSpace and subjected to descriptive analysis, in which numbers and percentages were used to describe the data statistically. The top 10 journals with the highest number of publications were identified, and their categories, Thomson Reuters journal rankings, 2023 impact factors (IF), and 5‐year IF were analyzed.

### 2.3 Co‐Citation Analysis

The data from “Full records and cited references” were imported into CiteSpace (V6.2, Drexel University, USA). The study configured parameters for a time span covering 1994–2023, with annual time slices. Co‐citation analysis targeted authors, countries (regions), institutions, journals and cited journals, authors and cited references, and keywords, utilizing cosine similarity for connection strength measurement. The screening criteria were applied to filter authors, countries (regions), institutions, journals and cited journals, authors, and cited references as per specified guidelines. The top number was set to 20; that is, the top 20 data points with the highest citation frequency or occurrence in each time period were selected. The keyword selection criterion was as follows: the top was set to five. The Pathfinder algorithm was used to trim co‐referenced maps.

### 2.4 Burst Detection Algorithm

A burst detection algorithm was employed to identify recent high‐impact countries (regions), institutions, and emerging keywords. The parameters were set with a time span from 1994 to 2023, utilizing annual time slices. The algorithm focused on analyzing bursts in countries (regions), institutions, and keywords, with connection strength assessed using cosine similarity. The screening criteria for countries (regions) and institutions were as follows: the number of top countries was set to 10; that is, the top 10 countries with the highest possible frequency or incidence in each time period were selected. The screening criterion for the keywords “top” was set to eight. The Pathfinder algorithm was used to trim co‐referenced maps.

### 2.5 Keyword Clustering Analysis

To distinguish between the research landscapes of various topics, keyword clustering utilized the logarithmic likelihood ratio (LLR). Keywords with LLR values ≥ 3.9 and *p* ≤ 0.05 were selected as significant indicators within each group. Clustering results were then analyzed using a time‐zone view to illustrate evolving trends over time.

### 2.6 Parameter Settings

The specific parameter settings of CiteSpace were as follows: Time‐slicing was chosen from 1994 to 2023, year by slice, and all options in the term source were selected; node types were selected one at a time; and selection criteria (g‐index, g^2^ ≤ *k* Si ≤ gci, *k* ∈ Z+, *k* = 25) [11]. Each node in the figure indicated an observation, including country, institution, co‐cited literature, and keywords.

## 3 Results

### 3.1 Annual Publications

**FIGURE 1:**
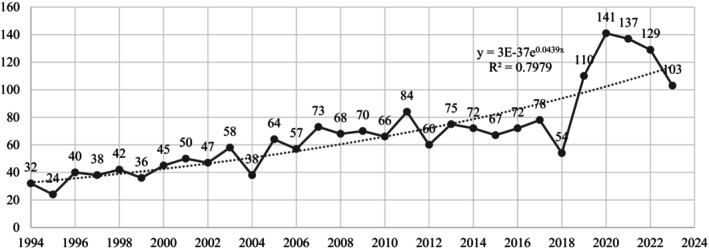
The number of annual publications.

Figure 1 illustrates the annual publication trends related to the Ilizarov technique. From 1994 to 2010, publications showed gradual growth, whereas from 2011 to 2023, they displayed increased variability. The peak occurred in 2020, with 141 articles published. Previous research has underscored the technique's significant impact on treating bone defects [12], fracture [13], and infected nonunion [14]. It has also influenced orthopedic research fields, contributing to accelerated studies since 2010. Predicting future trends, the function *Y* = 3E‐37e^0.0439*x*^ (*R* ^2^ = 0.7979), where *Y* represents annual publications and *x* denotes the year, reflects the strong growth trajectory in Ilizarov technique studies over the past three decades.

### 3.2 Types of Study

The types of studies included in this bibliometric analysis are presented in Table 1. A total of 11 types of studies were identified, with 1828 research articles accounting for 90.05%, 137 article reviews accounting for 6.75%, and 71 proceedings papers accounting for 3.50%. Most of the data were derived from original research. The most cited research article, reporting on osteomyelitis, antibiotic therapy, hyperbaric oxygen, joint infections, foot disease in patients with diabetes, and the use of the Ilizarov technique for the treatment of musculoskeletal infections, was published in *Clinical Orthopaedics And Related Research* in 2003 and received 429 citations [15]. The second most cited research article, reporting the advantages of lengthening over an intramedullary nail, including a decreased duration of external fixation, protection against refracture, and earlier rehabilitation [16], was published in the *Journal Of Bone And Joint Surgery‐American Volume* in 1997 and received 300 citations. The third most cited research article, which compared favorably with other methods of bone grafting and published accounts of the Ilizarov method, especially considering the large defect size in this series [17], was published in the *Journal of Orthopaedic Trauma* in 2000 and received 271 citations. The most cited review article, reporting on the use of the Ilizarov method in limb lengthening, skeletal reconstruction, and bone transport, was published in the *Journal Of Bone And Joint Surgery‐American Volume* in 1997 and received 248 citations [18].

**TABLE 1:** Study types of the included data.

No.	Study types	Amount	No.	Study types	Amount
1	Article	1828	7	Correction	3
2	Review article	137	8	Meeting abstract	3
3	Proceeding paper	71	9	Note	2
4	Editorial material	15	10	Biographical‐Item	1
5	Early access	12	11	Correction, Addition	1
6	Letter	11			

### 3.3 Author

**FIGURE 2:**
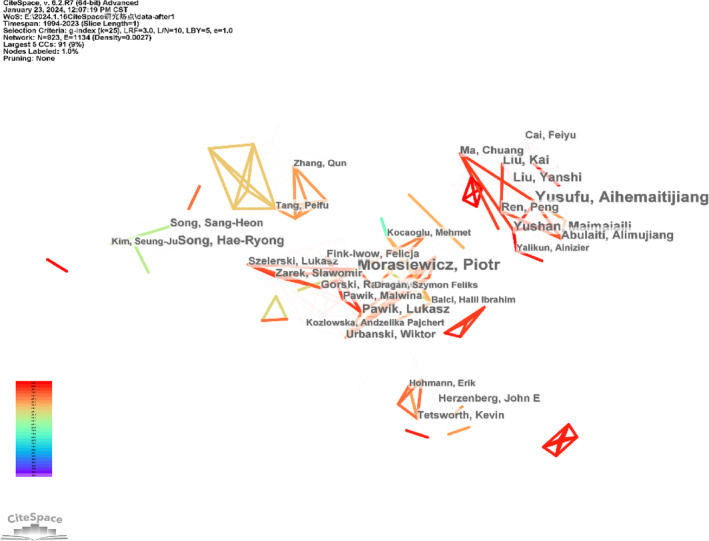
Map of the authors' cooperative relationships.

High‐impact authors in the Ilizarov technique field were identified using CiteSpace for co‐citation analysis, which produced a distribution map of co‐cited authors comprising 923 nodes and 1134 connections (Figure 2). According to co‐citation analysis theory, a prominent node indicates a key figure influencing the entire co‐cited network. The top five cited authors are Morasiewicz Piotr (26 citations) from the University of Opole in Poland, Aihemaitijiang Yusufu (26 citations) and Maimaiaili Yushan (13 citations) from The Fourth Affiliated Hospital of Xinjiang Medical University in China, and S Robert Rozbruch (21 citations) and Austin T Fragomen (20 citations) from the Hospital for Special Surgery in the USA.

### 3.4 Countries (Regions) and Institutions

**FIGURE 3:**
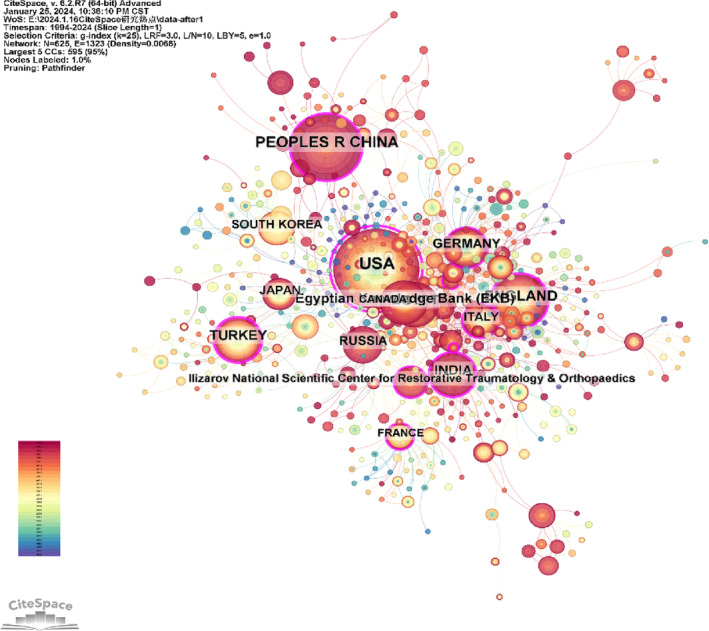
Map of countries' (regions') and institutions' cooperative relationships.

To identify prominent countries (regions) and institutions in the Ilizarov technique domain, CiteSpace was utilized to perform a co‐citation analysis. Figure 3 displays a network consisting of 625 nodes and 1323 connections among countries (regions) and institutions. The most frequently cited countries were the USA (366 citations), the People's Republic of China (232 citations), England (176 citations), Egypt (129 citations), and India (127 citations). The countries with the highest centrality included the USA (1.0), England (0.27), Turkey (0.23), the People's Republic of China (0.22), and India (0.22). The institutions receiving the most citations were the Egyptian Knowledge Bank (Egypt, 126 citations), Ilizarov National Scientific Center for Restorative Traumatology & Orthopaedics (Russia, 57 citations), Benha University (Egypt, 37 citations), Xinjiang Medical University (China, 29 citations), and Korea University (Korea, 29 citations). The institutions with significant centrality were the University of Leeds (England, 0.1), Benha University (Egypt, 0.07), Sinai Hospital of Baltimore (USA, 0.07), Ilizarov National Scientific Center for Restorative Traumatology & Orthopaedics (Russia, 0.06), and University of Texas System (USA, 0.05).

**FIGURE 4:**
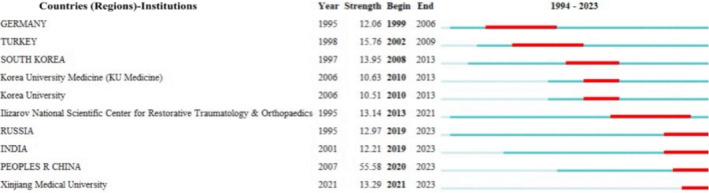
Top 10 countries (regions) and institutions with the strongest citation bursts. The blue line represents the time axis, and the red portion on the blue time axis represents the interval at which the burst was found, including the start year, end year, and burst duration.

To further investigate high‐impact countries (regions) and institutions recently, a burst detection algorithm was employed to analyze those receiving significant attention from the academic community and having frequent citations over a specified period. A higher burst value for a country (region) or institution indicates greater research output and impact during that time. Figure 4 lists the top 10 countries (regions) and institutions by burst values. The countries (regions) included the People's Republic of China (55.58), Turkey (15.76), South Korea (13.95), Russia (12.97), India (12.21), and Germany (12.06). The institutions are the Ilizarov National Scientific Center for Restorative Traumatology and Orthopaedics (13.14), Xinjiang Medical University (13.29), Korea University Medicine (10.63), and Korea University (10.51).

### 3.5 Journals and Cited Journals

This study identified 2030 articles published across 198 journals, with an average of 10.25 articles per journal. The fields of study are widely varied, indicating that the Ilizarov technique has been applied across numerous disciplines, exhibiting clear interdisciplinary attributes. The principal journals for publication included *Injury‐International Journal of the Care of the Injured* (117), *Journal of Pediatric Orthopaedics* (103), *Clinical Orthopaedics and Related Research* (97), *Journal of Orthopaedic Trauma* (78), *International Orthopaedics* (76), *Journal of Pediatric Orthopaedics Part B* (66), *Journal of Bone and Joint Surgery British Volume* (62), *Archives Orthopaedic and Trauma Surgery* (49), *Journal of Bone and Joint Surgery American Volume* (42), and *Acta Orthopaedica Belgica* (41), as detailed in Table 2. These top 10 journals published a total of 731 articles, constituting 36.00% of the total. Furthermore, the majority of Ilizarov technique studies were disseminated in six primary fields: critical care medicine, emergency medicine, orthopedics, surgery, pediatrics, and sports sciences. In terms of impact, the average IF of these journals in 2023 was 2.25, with 6 out of 10 journals having a 2023 IF exceeding 2 points, 7 out of 10 journals having a 5‐year IF over 2 points, and 3 journals achieving the top rank in the Thomson Reuters journal rankings. High‐quality reports on the Ilizarov technique are notably published in *Clinical Orthopaedics and Related Research* and *Journal of Bone and Joint Surgery American Volume*, both predominantly focusing on clinical studies in orthopedics and surgery.

**TABLE 2:** Top 10 journals with the most publications.

No.	Journal	Number of publications	Category (ranking)	Journal ranking of Thomson Reuters	2023 IF	5‐year IF
1	*Injury‐International Journal of the care of the injured*	118	Critical Care Medicine (27/35); Emergency Medicine (12/32); Orthopedics (36/86)	Q2	2.2	2.5
2	*Journal of Pediatric Orthopaedics*	103	Orthopedics (56/86); Pediatrics (88/130)	Q3	1.4	1.8
3	*Clinical Orthopaedics and Related Research*	97	Orthopedics (11/86); Surgery (27/213)	Q1	4.2	4.9
4	*Journal of Orthopaedic Trauma*	78	Orthopedics (42/86); Sport Sciences (49/87)	Q3	1.6	2.6
5	*International Orthopaedics*	76	Orthopedics (31/86)	Q2	2	2.6
6	*Journal of Pediatric Orthopaedics Part B*	66	Orthopedics (70/86); Pediatrics (49/87)	Q4	0.9	1.1
7	*Journal of Bone and Joint Surgery British Volume*	62	Orthopedics (5/72); Surgery (25/198)	Q1	3.3	3.5
8	*Archives Orthopaedic and Trauma Surgery*	49	Orthopedics (42/86); Surgery (97/213)	Q2	2	2.3
9	*Journal of Bone and Joint Surgery American Volume*	42	Orthopedics (5/86); Surgery (17/213)	Q1	4.4	5.4
10	*Acta Orthopaedica Belgica*	42	Orthopedics (85/86)	Q4	0.5	0.6

**FIGURE 5:**
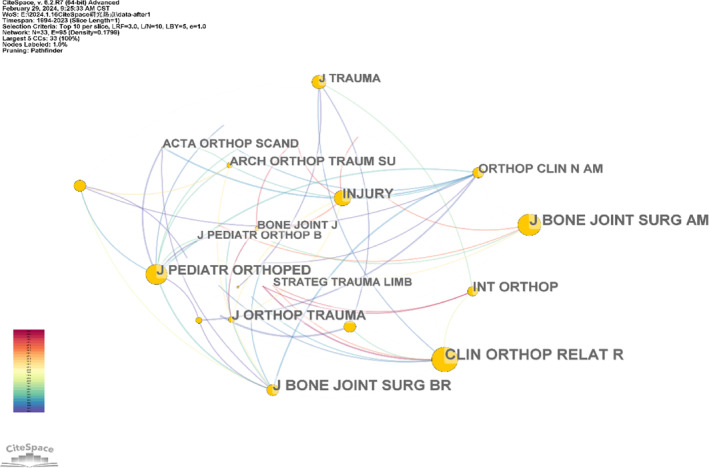
Map of journal co‐citations (*Clin Orthop Relat R*, *Clinical Orthopaedics and Related Research*; *J Bone Joint Surg AM*, *Journal of Bone and Joint Surgery American Volume*; *J Bone Joint Surg BM*, *Journal of Bone and Joint Surgery British Volume*; *J Orthop Trauma*, *Journal of Orthopaedic Trauma*; *J Pediatr Orthoped, Journal of Pediatric Orthopaedics*; *J trauma*, *Journal of Orthopaedic Trauma*; *Injury*, *Injury International Journal of the care of the injured*).

Figure 5 generated using CiteSpace, illustrates the co‐citation analysis of the cited journals, revealing 33 nodes and 95 connections. The size of each node correlates with the journal's co‐citation frequency. The top five journals in terms of co‐citation frequency include *Clinical Orthopaedics and Related Research* (1718 occurrences), *Journal of Bone and Joint Surgery American Volume* (1415 occurrences), *Journal of Bone and Joint Surgery British Volume* (1327 occurrences), *Journal of Pediatric Orthopaedics* (802 occurrences), and *Injury‐International Journal of the care of the injured* (763 occurrences). In terms of centrality, the leading journals are *Clinical Orthopaedics and Related Research* (0.60), *Journal of Bone and Joint Surgery American Volume* (0.47), *Journal of Pediatric Orthopaedics* (0.42), *Injury International Journal of the care of the injured* (0.26), and *Journal of Orthopaedic Trauma* (0.17).

### 3.6 Author and Cited Reference

In the reference co‐referencing network generated by CiteSpace, a crucial reference node is identified as one that connects multiple clusters. These key references, characterized by their high co‐citation frequency, occupy central positions in the knowledge flow network and serve as foundational elements in subject knowledge. The co‐citation network includes authors and cited references and comprises 1296 nodes and 3716 connections. This network includes four original articles and one review article, as listed in Table 3. Key studies relevant to the Ilizarov technique include the following: the suitability of the Ilizarov method for treating infected or non‐infected tibial bone defects [3]; a comparison between bone transport and acute shortening/lengthening for infected tibial segmental defects ranging from 3 to 10 cm [19]; an evaluation of the Masquelet technique versus Ilizarov bone transport in treating lower extremity bone defects after post‐traumatic osteomyelitis [20]; a comparison of radiological and functional outcomes between ring and rail fixators in patients with infected gaps (> 3 cm) non‐union of the tibia [21]; and the implementation of a treatment algorithm that incorporates four Ilizarov methods for addressing infected tibial non‐union, mobility of non‐union, and segmental defect size to determine the appropriate treatment choice [22]. These pivotal references form the basis of research concerning the Ilizarov technique.

**TABLE 3:** Top 5 authors and reference co‐citations.

No.	Frequency	Study types	Cited reference	Author (year of publication)
1	44	Review	Ilizarov bone transport and treatment of critical‐sized tibial bone defects: a narrative review	Kemal Aktuglu (2019) [3]
2	37	Article	Bone transport versus acute shortening for the management of infected tibial non‐unions with bone defects	Kevin Tetsworth (2017) [19]
3	33	Article	Masquelet technique versus Ilizarov bone transport for reconstruction of lower extremity bone defects following post‐traumatic osteomyelitis	Kai Tong (2017) [20]
4	24	Article	Prospective randomized comparison of ring versus rail fixator in infected gap non‐union of tibia treated with distraction osteogenesis	R. Rohilla (2016) [21]
5	24	Article	Ilizarov Treatment Protocols in the Management of Infected Non‐union of the Tibia	Martin McNally (2017) [22]

### 3.7 Analysis of Keyword Co‐Citation, Burst Value, Clustering, and Time Evolution

**FIGURE 6:**
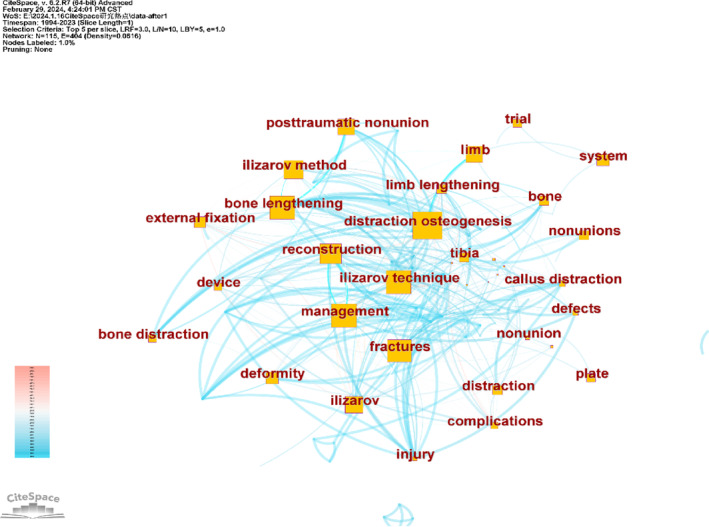
Map of co‐cited keywords.

Highly cited keywords reflect the focus of a discipline and indicate its sustainable development direction. Co‐citation analysis was performed to pinpoint prominent and pressing issues in the Ilizarov technique field. The keyword co‐citation network generated by CiteSpace, which features 115 nodes and 404 connections, is illustrated in Figure 6. The top 10 keywords by co‐citation frequency include management (374 times), Ilizarov technique (311 times), external fixation (248 times), distraction osteogenesis (248 times), reconstruction (186 times), Ilizarov method (146 times), complications (125 times), fractures (88 times), external fixator (50 times), and children (46 times). The top 10 keywords in terms of centrality are distraction osteogenesis (0.35), the Ilizarov technique (0.26), bone lengthening (0.26), management (0.25), fractures (0.23), reconstruction (0.18), the Ilizarov method (0.15), Ilizarov (0.12), limb (0.12), and post‐traumatic non‐union (0.12). Keywords such as management, the Ilizarov technique, distraction osteogenesis, and fractures are notable for their high co‐citation frequency and centrality. Keywords such as management, the Ilizarov technique, distraction osteogenesis, and fractures are notable for their high co‐citation frequency and centrality.

**FIGURE 7:**
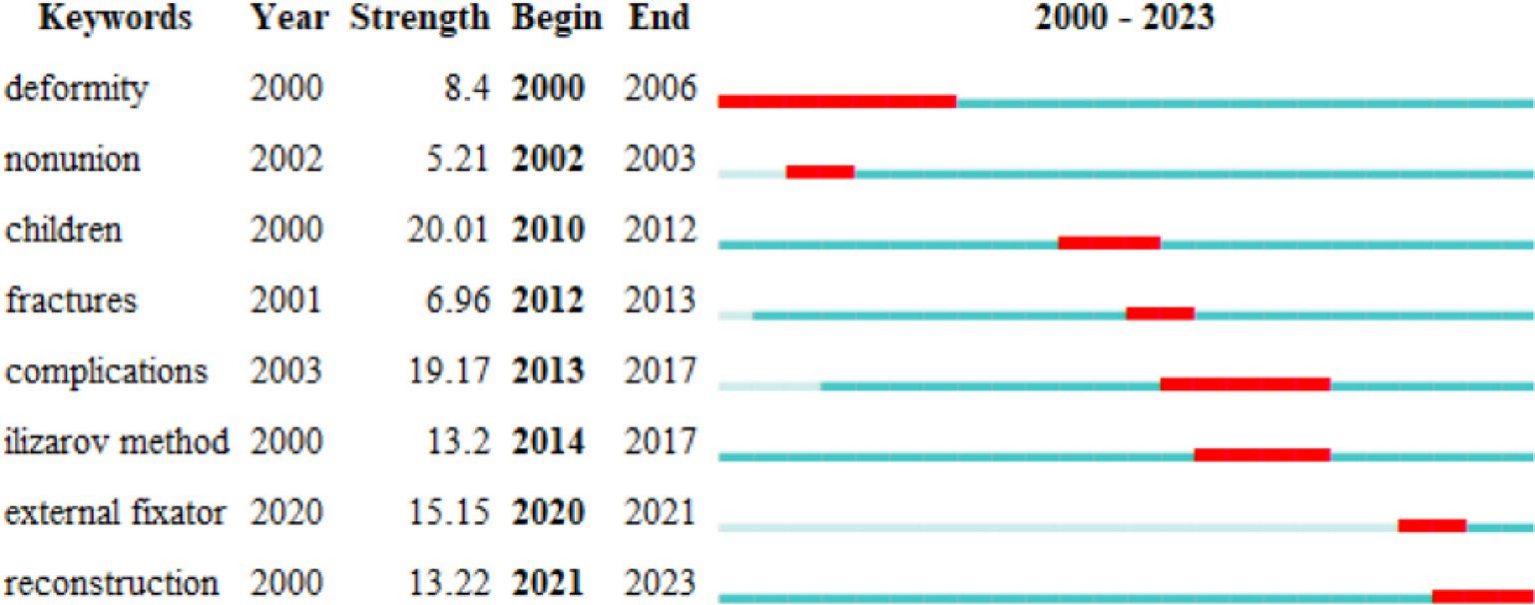
Top 8 keywords according to the burst values from 2000 to 2023. The blue line represents the time axis, and the red portion on the blue time axis represents the interval at which the burst was found, including the start year, end year, and burst duration.

To identify keywords that have recently garnered academic attention, we applied a burst detection algorithm. This method visualizes results in two dimensions: burst value and burst time. Keywords with high burst values indicate significant attention during specific time intervals, reflecting research frontiers. Figure 7 displays the top 8 keywords, with red nodes marking annual peaks. From 2000 to 2023, children, complications, external fixators, reconstruction, and the Ilizarov method exhibited strong bursts, with values of 20.01, 19.17, 15.15, 13.22, and 13.2, respectively, occurring predominantly within this period.

**FIGURE 8:**
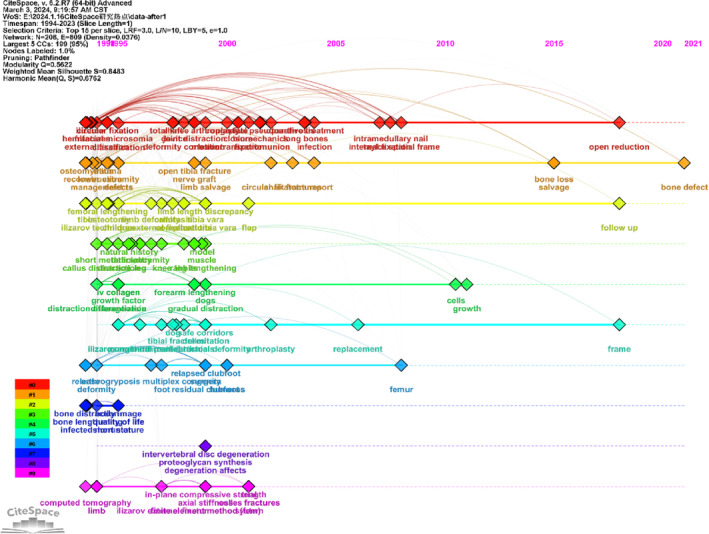
A timeline view for keywords associated with Ilizarov technique. The node's position on the horizontal axis represents the time when the keyword first appeared, and the node's size is positively correlated with the number of occurrences of the keywords. The lines between the nodes represent co‐occurrence relationships.

The maximum likelihood estimation method was initially introduced by CF Gauss, a German mathematician, in 1821. RA Fisher further explored its concepts in 1922, and it is now widely employed in cluster analysis to differentiate characteristics. Using LLR in keyword cluster analysis effectively reveals the internal characteristics of research subjects and provides robust evidence for predicting the evolution of research hotspots. Keywords with higher LLR values are more indicative within their clusters. We utilized LLR ≥ 3.9 and *p* ≤ 0.05 as screening criteria to categorize keywords into seven groups, as detailed in Table 4. To further explore the temporal evolution of these clusters, we conducted a time zone view analysis to illustrate dynamic changes in keywords across different periods, as shown in Figure 8.

**TABLE 4:** Cluster analysis of keywords.

Cluster ID	Keywords (positive likelihood ratio, P)	Content
1	External fixation (45.51, 1.0E‐4); bone transport (23.29, 1.0E‐4); fractures (14.47, 0.001); Taylor spatial frame (12.87, 0.001); internal fixation (11.64, 0.001)	External fixation in bone transport
2	Bone defect (25.73, 1.0E‐4); reconstruction (22.13, 1.0E‐4); masquelet technique (21.97, 1.0E‐4); deformity correction (18.25, 1.0E‐4); management (16.72, 1.0E‐4)	Management in bone defect
3	Ilizarov technique (52.33, 1.0E‐4); complications (36.5, 1.0E‐4); children (35.95, 1.0E‐4); external fixator (33.64, 1.0E‐4); osteotomy (16.54, 1.0E‐4)	Treatment of lower limb joint development deformity disease
4	Leg lengthening (19.72, 1.0E‐4); callus distraction (16.48, 1.0E‐4); tendon (14.52, 0.001); distraction (13.75, 0.001); rabbits (10.75, 0.005)	Limb extension therapy
5	Distraction osteogenesis (75.46, 1.0E‐4); dogs (9, 0.005); mesenchymal stem cells (9, 0.005); fractures (7.77, 0.01); growth factors (5.41, 0.05)	Treatment of the fracture and the non‐union after the fracture
6	Mechanical modulation (13.36, 0.001); immobilization (13.36, 0.001); intervertebral disc degeneration (13.36, 0.001); animal model (10.59, 0.005); compression (8.87, 0.005)	Mechanical of Ilizarov technique
7	Ilizarov external fixator (25.16, 1.0E‐4); axial stiffness (17.98, 1.0E‐4); in‐plane compressive strength (17.98, 1.0E‐4); system (14.18, 0.001); plantar pressure (8.96, 0.005)	System of Ilizarov external fixator

According to recent keywords in each cluster, terms such as open reduction, follow‐up, frame, and bone defect emerged after 2018. This suggests that future research directions will likely focus on the Ilizarov method for open reduction and bone defects, as well as follow‐up treatment and nursing strategies associated with the Ilizarov method, with continued in‐depth discussion anticipated.

## 4 Discussion

This study revealed that research on Ilizarov technology has grown rapidly since 1950 due to the increasing incidence of orthopedic diseases and injuries. The USA, England, Egypt, Benha University, and the Ilizarov National Scientific Center lead in research and collaboration, while China is a notable contributor. Among journals, *Clinical Orthopaedics and Related Research* stand out as a mainstream publication. Key themes include bone defects, external fixation, and use in children. Future trends should focus on device design, precision medicine, digital technology, and rehabilitation.

### 4.1 Trends in Ilizarov Technology Research

The inception of scholarly interest in Ilizarov technology can be traced back to 1950, with a steady rise in research activities. The exponentially increasing number of publications in this field led to 141 published articles in 2020, as recent articles continued to be built on from previous literature, with attempts to address unmet challenges or delve deeper into a specific research theme in this field. This flourishing research in Ilizarov technology can be attributed to the increasing prevalence of orthopedic diseases, including complex fractures, bone non‐union, bone defects, and osteomyelitis, resulting from the intensification of population aging and the growing number of accidental injuries, including traffic accidents [23, 24]. This market demand has provided ample room and impetus for the advancement of research in Ilizarov technology. However, the number of publications alone is not sufficiently informative to paint an overall picture of the involvement of countries, institutions, journals, and authors worldwide and is not reflective of traditional and contemporary research issues within this thematic area. This study addresses some of these viewpoint limitations by providing a comprehensive overall outlook of publication and envisioning prospects through the union of bibliometric and visualization analyzes.

The exponential function model in this study has demonstrated a prominent upward trend in publication number pertaining to the future, with an *R* ^2^ value of 0.7979. The *R* ^2^ value of 0.7979 indicates that the model explains approximately 79.79% of the variability in the data. An *R* ^2^ value between 0.5 and 0.9 may indicate that the model has a moderate degree of goodness of fit, which is still a relatively good result, but there may still be room for improvement [25]. This also means that about 20% of the annual publication volume changes remain unaccounted for in the model. This could be because the simple exponential function fails to capture certain factors, such as changes in research funding, technological advancements, or shifts in clinical practice.

### 4.2 Studies Focused on Ilizarov Technology Research

#### 4.2.1 Countries (Regions) and Institutions

The research momentum of different countries (regions) and institutions was analyzed via co‐citation analysis, including co‐authorship, citation frequency, centrality, and burst detection algorithm results, which suggested that these countries or regions had the highest weight in terms of literature volume, research quality, and collaboration, speaking to their leading positions in this field. Concerning citation frequency and centrality, the most productive and cooperative countries (regions) and institutions were the USA, England, and Egypt, along with Benha University and the Ilizarov National Scientific Center for Restorative Traumatology & Orthopaedics as the principal institutions. S. Robert Rozbruch led in publishing within the USA, contributing 10 articles primarily on the Ilizarov method as a valid approach for limb reconstruction in patients with bone tumors. However, an extended duration of external fixation was required [26]. Collaboration was observed between the USA and several countries, including China, Germany, Egypt, South Korea, England, and Russia, with Egypt and Russia showing the closest cooperative ties. International collaboration in previous research related to the Ilizarov technique was relatively active, especially in English‐speaking countries or regions or those with well‐established English education systems. Hence, language may be an important factor in promoting effective collaboration in this subject area [27].

An interesting observation was that China made substantial contributions to the field, reflected by its second position in total citation frequency and fourth in total centrality analysis, but first in citation burst strength among countries or regions worldwide. This could imply that papers from China were of greater quality and impact. More interestingly, the citation burst values of the USA, England, and Egypt did not fall into the top six. It could also be that articles from these countries or regions had a relatively smaller impact due to the larger scale of publication volume [28]. A lower citation burst strength was not directly related to lower academic impact [29]. In comparison, the high burst value may be attributed to the significance and innovation of the research findings or their high relevance to current hot topics. Recently, China has emerged as a notable player in the Ilizarov technology field, achieving its highest burst value of about 55.58 between 2020 and 2023. In terms of citation numbers, in 2022, two institutions in China and Australia reviewed 3D printing technology based on computer technology, aiming to develop personalized artificial scaffolds that accurately fit bone defects, which has received widespread attention [30].

#### 4.2.2 Journals

The research impacts of specific journals in the field of Ilizarov technology were studied via the number of publications, impact factor, co‐citation frequency and centrality analysis. The *Injury‐International Journal of the Care of the Injured* had the highest publication volume. *Clinical Orthopaedics and Related Research* was also a high‐achieving journal, as its publication volume ranked third, and its co‐citation frequency ranked first. Interestingly, *Injury‐International Journal of the Care of the Injured* with the highest published volume disappeared in the top three co‐citations. This demonstrates that there was no direct correlation between journals with high publication outputs and those that garner significant attention from the academic community [31]. Journals with substantial publication volumes may not necessarily be central within a specific research field; instead, the distribution of such journals is discerned through the analysis of “co‐cited journals” within the academic community [27]. The co‐citation frequency serves as an indicator of a journal's quality and influence [27]. Journals that exhibit high co‐citation frequencies are commonly recognized as mainstream within the academic sphere [32]. *Clinical Orthopedics and Related Research*, with an IF of 4.2 in 2023 and an annual publication count of approximately 600, emerged as a mainstream journal in the field, ranking in the top quartile for orthopedics and surgery. This journal focused primarily on diagnosing and treating musculoskeletal conditions, publishing research that significantly contributes to the foundational understanding of the Ilizarov technique.

The *Journal of Bone and Joint Surgery British Volume* had the third highest co‐citation frequency and was absent from the top five centrality rankings. The mismatch between top journal's centrality and co‐citation rankings may reflect the level of unique or novel arguments presented in the published literature [33]. The journal has a high co‐citation ranking but a low centrality ranking, which may suggest that the journal has published many studies closely related to other literature in the field of the Ilizarov technique; however, these studies may lack innovation or uniqueness. Conversely, when the journal has a high centrality ranking but a low co‐citation ranking, it may indicate that the journal has published some unique or novel arguments in the Ilizarov technique, which have not yet been widely accepted or formed close connections with other literature. Thus, journals with high co‐citation link strength may be associated with more new discussions.

Morasiewicz Piotr, Aihemaitijiang Yusufu, and Maimaiaili Yushan are the top three authors with the highest co‐citation frequency in this field. The top 20 authors can be considered pioneers in the Ilizarov technique. Future research may significantly impact the development of this field and should be closely monitored to keep up with the latest advancements.

#### 4.2.3 Future Prospects

Highly cited keywords reflect the focus of a discipline and indicate its sustainable development direction [27]. Co‐citation analysis was performed to pinpoint prominent and pressing issues in the Ilizarov technique field. The themes in specific fields coordinated by publication year open a window for future research directions. From 2000 to 2023, children, complications, external fixators, reconstruction, and the Ilizarov method exhibited strong bursts, predominantly occurring within this period. Ilizarov technology has evolved from its original circular external fixation system to include unilateral single‐plane, multilateral multiplane external fixation, and intelligent multiaxis external fixation systems. Initially, used for fracture fixation, it now addresses trauma sequelae, osteomyelitis, limb deformity correction, and lower limb ischemic disease. Recent reports highlight its successful application in treating advanced knee tuberculosis [34] and severe knee flexion contracture due to rheumatoid arthritis in children [35], indicating that children are a current research focus in Ilizarov treatment. Ilizarov's treatment has significant social implications in many countries because of its transformative yet resource‐intensive nature. The prevailing themes in this field can be broadly categorized into seven clusters, each enclosing a family of subthemes. Key research focuses on bone defects, including external fixation in bone transport, management of bone defects, treatment of lower limb joint development deformities, limb extension therapy, treatment of fractures and non‐union, mechanical treatment via the Ilizarov technique, and use of the Ilizarov external fixator system. The findings of many of the present meta‐analyzes indicate that the Ilizarov technique is a successful treatment option for bone defects [36, 37]. This method has demonstrated efficacy in achieving the expected clinical outcomes.

Ilizarov technology, also known as distraction osteogenesis, is a surgical technique used to lengthen or correct bone deformities [38]. It involves the gradual separation of bone segments over time, allowing new bone to form in the gap [38]. The signaling pathways involved in this process are complex and multifaceted and involve both mechanical and biological stimuli. The mechanical tension created by the gradual destruction of bone segments activates mechanotransduction pathways, converting mechanical stimuli into biochemical signals that regulate bone growth and remodeling. The molecular mechanisms underlying Ilizarov technology involve a delicate balance between bone resorption and bone formation. Osteoclast activation initiates bone resorption, with RANKL (receptor activator of nuclear factor‐κB ligand) produced by osteoblasts and stromal cells binding to RANK receptors on osteoclast precursors [39]. As the gap between bone segments widens, BMSCs differentiate into osteoblasts, driven by BMPs, TGF‐β, and IGF‐1. ECM remodeling plays a crucial role in supporting cell adhesion, migration, and differentiation during bone regeneration [39].

Future research trends in Ilizarov technology may revolutionize the field of orthopedic treatment, particularly in the context of limb lengthening and deformity correction. Advancements are anticipated to focus on several key areas: optimization of device design, with researchers likely to explore innovative designs that increase patient comfort, reduce the risk of complications, and improve ease of use for both patients and clinicians; precision medicine and personalized treatment plans, with future studies aiming to tailor Ilizarov treatment plans on the basis of patients' genetic profiles, biomechanical characteristics, and specific medical histories; integration of digital technologies such as 3D printing, CAD, and AI to increase the precision and customization of Ilizarov devices; and enhanced rehabilitation and post‐treatment care, addressing traditional challenges associated with Ilizarov technology, such as prolonged treatment durations, frequent needle tract infections, and increased fracture recurrence risks, with future research focusing on optimizing protocols and care pathways to maximize functional recovery and minimize long‐term complications. Overall, the future of Ilizarov technology holds great promise for advancing orthopedic care, with a strong emphasis on innovation, personalization, and the integration of cutting‐edge technologies to improve patient outcomes and experiences.

### 4.3 Strengths and Limitations

We conducted a comprehensive and objective search of Ilizarov‐related literature using WOS, providing insights into the research characteristics and citations of articles published in the field of Ilizarov. However, this study has many limitations. First, only English articles in WOS were included in this study, and unpublished works and articles in languages other than English were excluded. Second, bibliometric data are subject to change over time, and indexing delays can contribute to partial variations in our results. Third, we did not delve into the content of individual articles to differentiate whether they were cited for their positive contributions, negative impacts, or poor quality; rather, we included all articles without distinction. Additionally, we did not consider whether authors cited papers based on personal preferences for particular journals. These are all possible sources of bias.

## 5 Conclusions

This study highlights the current status and trends in Ilizarov technology worldwide. The USA is the leading country in terms of total citation frequency and centrality. Concerning citation burst strength, China has also made significant contributions to this field. The journal *Injury International Journal of the Care of the Injured* published the greatest number of papers on this topic. It can be predicted that the number of papers will continue to rise in the next decade. Future advancements in Ilizarov technology hinge not only on enhancing fundamental biological theories but also on advancing application concepts and device development. The integration of external fixation systems with information technology and the emergence of new composite treatment strategies are gradually addressing traditional challenges associated with Ilizarov technology, such as prolonged treatment durations, frequent needle tract infections, and increased fracture recurrence risks. However, future research on follow‐up home‐based rehabilitation guidance for Ilizarov technique treatment is still needed.

## Author Contributions

Xinxin Wang contributed to the design of the study, was responsible for the collection and retrieval of data, contributed to data analysis and interpretation, drafted the manuscript. Wenxia Wang and Xiaoyan Zhi also contributed to data collection. Fei Lu conceptualized and designed the study, supervised all aspects of the study, critically reviewed and revised the manuscript, and approved the final manuscript as submitted.

## Ethics Statement

The authors have nothing to report.

## Consent

The authors have nothing to report.

## Conflicts of Interest

The authors declare no conflicts of interest.

## Data Availability

Our data or material may be available from corresponding author or first author upon reasonable request.
